# Pathogenic Role of Store-Operated and Receptor-Operated Ca^2+^ Channels in Pulmonary Arterial Hypertension

**DOI:** 10.1155/2012/951497

**Published:** 2012-09-27

**Authors:** Ruby A. Fernandez, Premanand Sundivakkam, Kimberly A. Smith, Amy S. Zeifman, Abigail R. Drennan, Jason X.-J. Yuan

**Affiliations:** ^1^Section of Pulmonary, Critical Care, Sleep and Allergy Medicine, Department of Pharmacology, Institute for Personalized Respiratory Medicine, Center for Cardiovascular Research, University of Illinois at Chicago, Chicago, IL 60612, USA; ^2^Department of Medicine, University of Illinois at Chicago, Chicago, IL 60612, USA

## Abstract

Pulmonary circulation is an important circulatory system in which the body brings in oxygen. Pulmonary arterial hypertension (PAH) is a progressive and fatal disease that predominantly affects women. Sustained pulmonary vasoconstriction, excessive pulmonary vascular remodeling, *in situ* thrombosis, and increased pulmonary vascular stiffness are the major causes for the elevated pulmonary vascular resistance (PVR) in patients with PAH. The elevated PVR causes an increase in afterload in the right ventricle, leading to right ventricular hypertrophy, right heart failure, and eventually death. Understanding the pathogenic mechanisms of PAH is important for developing more effective therapeutic approach for the disease. An increase in cytosolic free Ca^2+^ concentration ([Ca^2+^]_cyt_) in pulmonary arterial smooth muscle cells (PASMC) is a major trigger for pulmonary vasoconstriction and an important stimulus for PASMC migration and proliferation which lead to pulmonary vascular wall thickening and remodeling. It is thus pertinent to define the pathogenic role of Ca^2+^ signaling in pulmonary vasoconstriction and PASMC proliferation to develop new therapies for PAH. [Ca^2+^]_cyt_ in PASMC is increased by Ca^2+^ influx through Ca^2+^ channels in the plasma membrane and by Ca^2+^ release or mobilization from the intracellular stores, such as sarcoplasmic reticulum (SR) or endoplasmic reticulum (ER). There are two Ca^2+^ entry pathways, voltage-dependent Ca^2+^ influx through voltage-dependent Ca^2+^ channels (VDCC) and voltage-independent Ca^2+^ influx through store-operated Ca^2+^ channels (SOC) and receptor-operated Ca^2+^ channels (ROC). This paper will focus on the potential role of VDCC, SOC, and ROC in the development and progression of sustained pulmonary vasoconstriction and excessive pulmonary vascular remodeling in PAH.

## 1. Introduction

The only organ in the body to receive the entire cardiac output (CO) at one time is the lung. To receive a high flow of volume from the entire CO, the pulmonary circulatory system must maintain a low-resistance and low-pressure system to carry blood to the pulmonary capillaries. Deoxygenated venous blood flows through the pulmonary artery to the pulmonary capillaries where oxygen and carbon dioxide gas exchange occurs. Pulmonary hypertension (PH) is a severe chronic disorder that affects the pulmonary circulatory system. This disorder is often a deadly hemodynamic irregularity that may be idiopathic, heritable, or secondary to other diseases such as chronic obstructive pulmonary disease (COPD). Pulmonary arteries are thin and have low myogenic tone compared to systemic arteries. Therefore, pulmonary arteries rely on arterial distension and recruitment for decreasing pulmonary vascular resistance (PVR) after increased blood flow or increased CO (e.g., during heavy exercise). In the lungs, increased PVR is the major cause for the development of PH. An increased PVR results in increased RV afterload which leads to right heart failure and eventually death [1]. 

Pulmonary arterial pressure (PAP) varies over a lifetime. During early childhood through the age of about 50 years, maximum PAP is near 20 mm Hg. Pulmonary arterial hypertension (PAH) is clinically defined as a resting mean pulmonary arterial pressure (mPAP) greater than or equal to 25 mm Hg at rest, or an mPAP greater than or equal to 30 mm Hg during exercise [2]. PAP is the product of CO and PVR, and the equation is as follows (PAP = CO × PVR). In this equation PVR, is the vascular resistance from the whole lung (PVR_arteries_ + PVR_capillaries_ + PVR_veins_) [3]. In healthy individuals, the arteries, capillaries, and veins have a compensative elastic mechanism, which results in an increase in the cross-sectional area of the pulmonary vascular bed. Therefore, exercise causes a marginal change in PAP, following increased CO. Conversely, patients with pulmonary hypertension at rest (no increased CO) have increased levels of PAP, due to an increase in PVR from the arteries, capillaries, and veins. PVR is related to the flow of liquid through a cylindrical structure using the Poiseuille equation (PVR = (8*Lη*/*π*) × (1/*r* ^4^)), where *L* is the length of the artery, *r* is the inner radius, and *η* is the viscosity coefficient of the blood. Consequently, even small changes in the inner radius of the vessels can significantly change PVR, and therefore, PAP. 

PAH is a disease that is often misdiagnosed during routine medical examination. There are subclassifications of PH established by the World Symposium of Pulmonary Hypertension to distinguish PAH from other forms of PH (Table 1) [4, 5]. Physicians additionally use two classification systems set forth by the New York Heart Association, in addition to the WHO classification system, to diagnose the severity of PH. Tables 1 and 2 show the different criteria that explain the clinical, physical, and functional characteristics of PH [6]. A noninvasive initial screening is achieved by estimating PAP levels through echocardiography; however, the standard for clinical diagnosis of PH is by right heart catheterization [2]. Screening done by the recent REHAP Registry in Spain showed a 3.4 : 1 incidence of PAH in women to men, with an average age of 45 ± 17 years [7]. Even though various diagnoses exist, PAH is often misdiagnosed for other related diseases, such as congenital heart disease, emphysema, or pulmonary embolism.

## 2. Pathology and Pathophysiology of PAH

**Figure 1:**
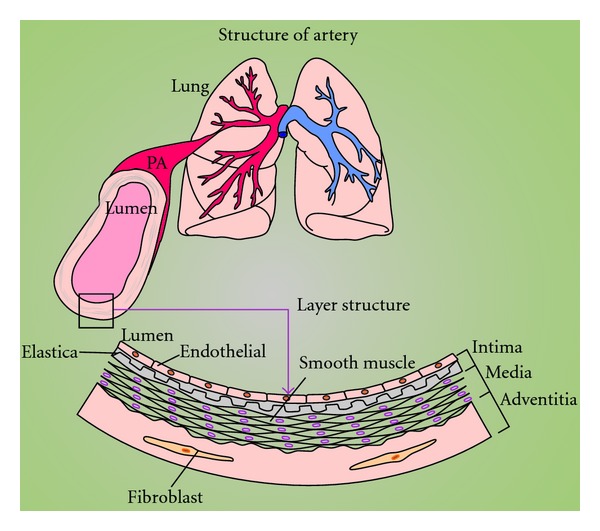
Structure of the pulmonary vasculature. Schematic demonstration of lung and pulmonary artery along with zoomed diagram of 3-layered structure of the artery.

**Figure 2:**
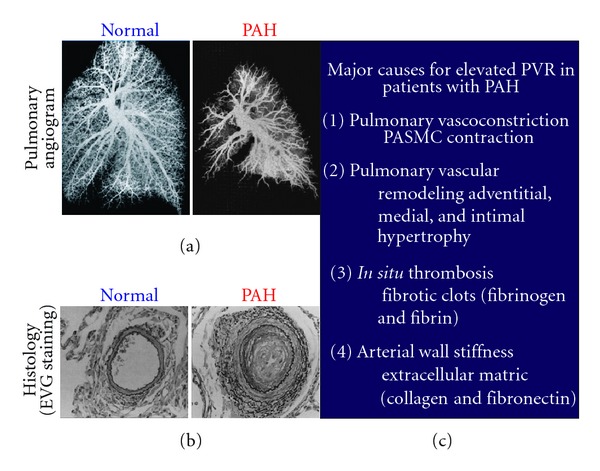
Major causes for elevated PVR in patients with PAH. (a) Pulmonary angiogram from a healthy (normal) patient compared to a PAH patient, depicting the loss (occlusion) of pulmonary arteries. (b) Pulmonary EVG histology staining of cross-sections of a healthy patient compared to a PAH patient, depicting vascular remodeling and medial and intimal layer thickening. (c) Listed are the four major causes of elevated PVR in PAH patients [8].

There are 15 orders of branching in the pulmonary arteries between the main pulmonary artery and the capillaries in the human lung [9]. The pulmonary artery is formed by three layers, inner intima (pulmonary arterial endothelial cells), media (pulmonary arterial smooth muscle cells [PASMC]), and outer adventitia (fibroblasts) (Figure 1). As the orders of branching increase, the diameter of the artery decreases. Vascular remodeling occurs throughout all branches causing thickening of the all layers, thus decreasing the radius and increasing PAP (Figure 2(b)) throughout the pulmonary arteries. The adventitial compartment of the vessel walls has been shown to undergo early structural changes following exposure to hypoxia. Proliferation of fibroblasts has been shown to sustain and exceed that of PAEC or PASMC in these models [10]. Endothelial, smooth muscle cells and fibroblast in the vascular wall play a specific role in the response to injury. However, fibroblasts are a relatively ill-defined cell that, at least compared to the SMC, exhibits few specific cellular markers. Thus, this paper will specifically focus on PASMC.

The pathogenesis of PAH is attributed to the collective effects of vascular remodeling, persistent vasoconstriction, *in situ* thrombosis, and arterial wall stiffening, together these attributes increase PVR leading to right heart failure [11, 12]. Features of pulmonary vascular remodeling in PH include medial and intimal cell layer thickening and intimal lesions that occlude (thrombosis) the artery which are attributed to PASMC abnormalities [13]. Angiograms and histology images from PAH patients reveal both remodeling and thrombosis (Figure 2). The angiogram in Figure 2 depicts narrowing or occlusion of the arteries. These images were obtained by injection of silicone into the main pulmonary artery. In the angiograms of PAH patients, the silicon is unable to reach smaller arteries and therefore is not depicted (Figure 2(a)). This is not a loss of arteries but rather a representation of the vascular remodeling, vasoconstriction, and *in situ* thrombosis causing occlusions in the pulmonary artery [8].

Pulmonary vascular remodeling is mainly caused by increased cell proliferation (and/or growth) and/or decreased cell apoptosis. *In vivo*, a balance of apoptosis and proliferation of pulmonary vascular wall cells sustains the thickness and tissue mass of the pulmonary arterial walls. Disturbance of this balance in favor of proliferation results in pulmonary arterial wall thickening, intraluminal narrowing, and eventually leading to increased PVR and thus elevated PAP. Experimental models are used to understand the importance of remodeling and vasoconstriction in the development of PAH. Hypoxic pulmonary vasoconstriction (HPV) is an important physiological mechanism that optimizes ventilation-perfusion matching and pulmonary gas exchange by diverting blood flow from poorly ventilated areas of the lung to well-ventilated area to maximize oxygenation [14]. In rats, hypoxic exposure results in rapid structural remodeling in pulmonary arteries indicating an imbalance of apoptosis and proliferation of smooth muscle cells and fibroblasts. After exposure to hypoxia, smooth muscle cell proliferation was evident, indicated by increasing amounts of cells in mitosis [15]. Previous studies suggest that decreased apoptosis is also associated with the development and maintenance of severe PH [14]. More pulmonary arterial smooth muscle cells (PASMCs) are in the synthetic (or proliferative) phenotype in animals with hypoxia-induced pulmonary hypertension (HPH) than in normoxic control animals; the increased PASMC proliferation is often associated with vascular wall hypertrophy and increased matrix production [13]. There are two different subtypes of smooth muscle cells (SMCs) that are present in the medial layer: a contractile phenotype and proliferative phenotype. In healthy adults, SMCs are in the contractile phenotype with abundant and well-organized thick and thin filaments found in the cytoplasm [16]. During embryogenesis or vascular injury, SMCs are shown to dedifferentiate into proliferative phenotype with a decreased amount of contractile proteins and increased amount of endoplasmic reticulum, ribosomes, and Golgi [16, 17]. The quiescent contractile phenotype is pertinent to the management of vascular tone and its regulation through endothelial factors that affect vascular resistance. The dedifferentiated proliferative phenotype is important in vascular thickening and remodeling such that it is involved in the pathogenesis of PAH [18, 19]. It is believed that both vasoconstriction and cellular proliferation share a common pathway involving different signaling processes. One of the common pathways is dependent, at least in part, on the regulation of Ca^2+^ homeostasis in PASMC. 

In addition to increased vascular remodeling, PH is also characterized by increased vasoconstriction. Vasoconstriction refers to an increase in tensile force, which translates to the narrowing of the lumen of the vessel. Causes of increased sustained vasoconstriction include increased [Ca^2+^]_cyt_ in PASMC, which leads to smooth muscle contraction by both Ca^2+^-dependent and Ca^2+^-independent mechanisms. Ca^2+^ directly activates myosin light chain kinase (MLCK) leading to contraction and migration. Additionally, an increase in [Ca^2+^]_cyt_ stimulates Ca^2+^-dependent signal transduction proteins. In a healthy individual, the pulmonary circulatory system has low resistance and easily dilates to accommodate the entire CO with a marginal change in pressure. In contrast to the systemic circulatory system, which has higher resistance and pressure, hypoxia causes the pulmonary arteries to constrict while the systemic arteries to dilate [20]. Hypoxia-induced vasoconstriction has been widely known to be associated with increased levels of [Ca^2+^]_cyt_ in PASMC, increased contractile elements in lung tissue, and enhanced sensitivity of contractile proteins. Several studies in the past have demonstrated that the amplitude of contraction can be affected by any change in the ratio of myosin light chain kinase (MLCK): MLCP activity. MLCK activity is dependent on Ca^2+^ calmodulin; hence, the rise in [Ca^2+^]_cyt_ is the primary determinant of smooth muscle contraction. The increase in sensitivity of smooth muscle contractility to [Ca^2+^]_cyt_ (i.e., Ca^2+^ sensitization) may be caused by inhibition of MLCP activity. It has been found that the predominant pathological cause in idiopathic pulmonary arterial hypertension (IPAH) in patients is a loss of vascular compliance and an increase in PVR due to pulmonary vascular remodeling and vasoconstriction [21]. 

*In situ* thrombosis may partially or completely occlude the pulmonary artery contributing to an increased PVR in patients with PAH. Endothelial cell dysfunction as well as interaction with growth factors and platelets causes obliterative pulmonary hypertension. This type of pulmonary hypertension is caused by a procoagulant environment within the pulmonary vascular bed [3]. Additionally, an important contributor to PAH is increased pulmonary vascular wall stiffness due to increased extracellular matrix. Hypoxic animal models have increased adventitial thickening, excessive extracellular matrix proteins (type I collagen, cellular fibronectin, and tenascin-C [TN-C]), and myofibroblast accumulation/differentiation compared to their normoxic control [22]. 

In summary, sustained pulmonary vasoconstriction, excessive pulmonary vascular remodeling, *in situ* thrombosis and increased pulmonary vascular wall stiffness are the four major causes of elevated PVR and PAP which lead to PAH. This paper explores the important role of Ca^2+^ and specific Ca^2+^ channels that are involved in the Ca^2+^ regulation in PASMC leading to the initiation and progression of PAH. 

## 3. Ca^2+^ Signaling in Pulmonary Vasoconstriction and PASMC Proliferation

**Figure 3:**
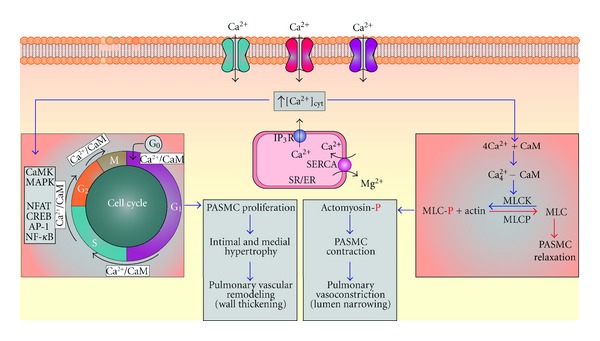
Increased levels of [Ca^2+^]_cyt_ in PASMC are required for pulmonary vascular remodeling and pulmonary vasoconstriction. When levels of [Ca^2+^]_cyt_ increase due to influx through various Ca^2+^ channels in the plasma membrane and by depletion of SR/ER stores, Ca^2+^ can bind to calmodulin (CaM) leading to PASMC contraction by activating myosin light chain kinase (MLCK) causing phosphorylation of MLC, resulting in a sliding motion of the actomyosin complex leading to contraction. Additionally, Ca^2+^ activates intracellular Ca^2+^-dependent signal transduction proteins such as CaM kinase (CaMK) and mitogen-activated protein kinase (MAPK), as well as activating other transcription factors (nuclear factor of activated T cells (NFAT, cAMP response element binding protein (CREB), activator protein-1 (AP-1), and nuclear factor (NF-*κ*B) (that trigger PAMSC to enter the cell cycle from a quiescent differentiated state leading to proliferation).

**Figure 4:**
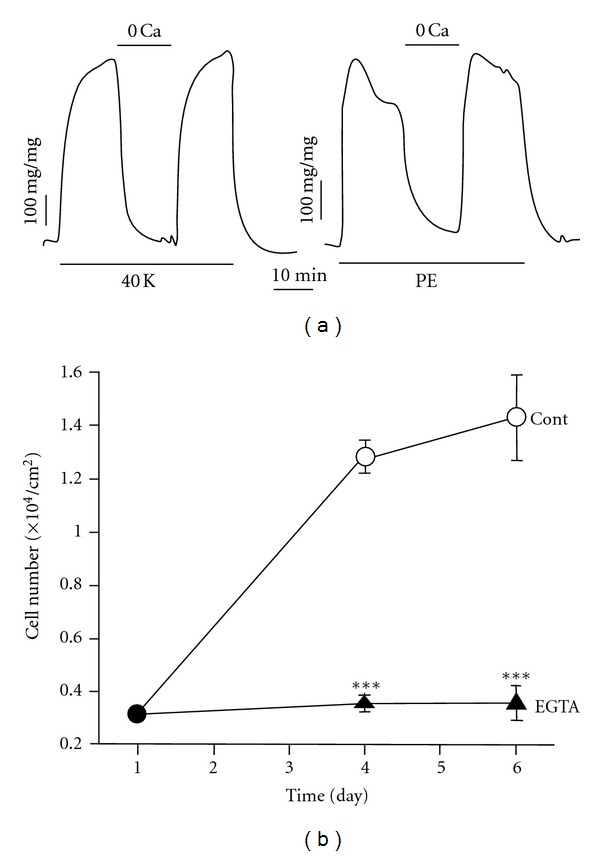
Ca^2+^ is required for PASMC contraction and PASMC proliferation. (a) Extracellular Ca^2+^ is required for pulmonary vasoconstriction induced by 40 mM K^+^ (40 K) and phenylephrine (PE). Ca^2+^-free solution was applied to the vessels when 40 mM K^+^- or PE-mediated contraction reached a plateau. Data showing active tension induced by 40 mM K^+^ or 2 *μ*M PE before (control), during (0 Ca), and after (recovery) application of Ca^2+^-free solution [25]. (b) Inhibition of rat PASMC growth by chelating extracellular Ca^2+^. Cells were cultured in 10% FBS-DMEM in the absence (Cont) and presence of 2 mM EGTA. Viable cell numbers were determined 1, 4, and 6 days after cells were plated. Data are means ± SE (*n* = 12 dishes of cells/group). **P < 0.01, ***P < 0.001 versus control [31].

Different forms of PH share common features of abnormalities in pulmonary vascular function, vascular cell proliferation, and remodeling, suggesting that they share essential downstream signaling mechanisms associated with disease progression. Intracellular Ca^2+^ signaling is highly critical for numerous physiological and pathophysiological processes in PASMC specifically those associated with pulmonary vasoconstriction, vascular cell proliferation, and remodeling [23, 24]. Several intriguing investigations have led to the understanding of mechanisms involved in the regulation of [Ca^2+^]_cyt_ in PASMC. Vascular smooth muscle contraction decreases the radius of pulmonary blood vessels, leading to sustained vasoconstriction and to increased PVR. The mechanism of contraction in pulmonary vascular smooth muscle is different than that of striated cardiac or skeletal muscles. Skeletal muscles undergo fast contractions, which involve Ca^2+^ binding to troponin. However, in PASMC slow, tonic contractions are initiated through changes in [Ca^2+^]_cyt_ that cause a Ca^2+^-activated phosphorylation of myosin. When [Ca^2+^]_cyt_ rises, Ca^2+^ binds to calmodulin (CaM) and activates myosin light chain kinase (MLCK) which then phosphorylates the myosin light chain. Phosphorylation of MLC increases myosin ATPase activity that hydrolyzes ATP to release energy. The subsequent cycling of the myosin cross-bridges produces a sliding motion of myosin-actin filaments and results in contraction of the smooth muscles (Figure 3) [24]. Our previous data show that removal of extracellular Ca^2+^ blocks the high K^+^-induced and phenylephrine- (PE-) induced contraction in isolated rat pulmonary arterial rings, illustrating that Ca^2+^ influx is necessary for smooth muscle contraction (Figure 4(a)) [25]. PAH patients have sustained pulmonary vasoconstriction, a major contributor to increased PVR and PAP. 

In addition to causing PASMC contraction, increased [Ca^2+^]_cyt_ is also important for cell proliferation and gene expression [23, 26]. Activation of Ca^2+^-sensitive signal transduction proteins (such as CaM kinase and mitogen-activated protein kinase) and transcription factors (such as NFAT, CREB, AP-1, and NF-*κ*B) can stimulate cell proliferation (Figure 3) [27–30]. Increased [Ca^2+^]_cyt_ is an important stimulus for cellular proliferation (Figure 4(b)) [31].In PASMC specifically, maintaining Ca^2+^ in the sarcoplasmic reticulum/endoplasmic reticulum (SR/ER) is vital for cell growth [32]. It has been shown that in the presence of serum and growth factors, the removal of extracellular Ca^2+^ and the depletion of ER-stored Ca^2+^ inhibit proliferation of PASMC, indicating the crucial role for Ca^2+^in the cell cycle progression and growth (Figure 4(b)). Ca^2+^ influx mechanisms contributing to the maintenance of [Ca^2+^]_cyt_ play a central role during several phases of the cell cycle. Growth factor-induced increase in [Ca^2+^]_cyt_ via Ca^2+^ release from the intracellular stores and Ca^2+^ entry from the extracellular space stimulates quiescent cells that are in the G_0_ phase to enter the cell cycle (G_1_) [33]. Thus, the maintenance of [Ca^2+^]_cyt_ is essential for cell proliferation.

## 4. Regulation of Cytosolic [Ca^2+^] in Normal PASMC and the Role of Ca^2+^ in the Pathology and Pathophysiology of PAH

Intracellular Ca^2+^-dependent pathways play an important role in the regulation of numerous physiological and pathophysiological processes in PASMC such as contraction, proliferation, and migration [34]. Studies indicate significant changes in the expression of ion channels resulting in marked alterations in Ca^2+^ homeostasis, such as membrane depolarization, increase in Ca^2+^ entry, elevation of resting [Ca^2+^]_cyt_, and contractile Ca^2+^ sensitivity, altogether contributing to vasoconstriction, and thus leading to PH. Ca^2+^ influx in PASMC is mainly regulated by voltage-dependent Ca^2+^ channels and voltage-independent nonselective cation channels. 

### 4.1. Voltage-Dependent Ca^2+^ Channels in PASMC

**Figure 5:**
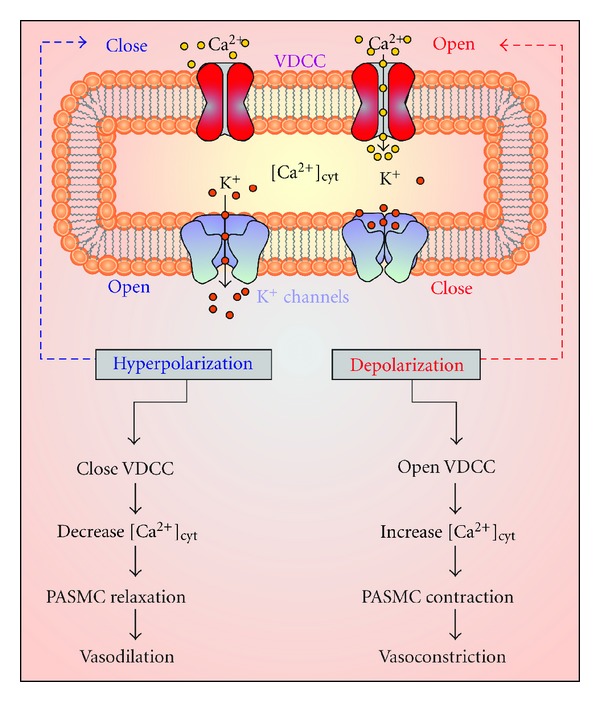
*K* ^+^ channels play a role in membrane depolarization and vasoconstriction. *K* ^+^ channels remain closed until stimulated to open, after stimulation, *K* ^+^ channels open causing membrane depolarization and leading to the opening of voltage-dependent Ca^2+^ channels (VDCC) and contribute to increased levels of [Ca^2+^]_cyt_ causing vasoconstriction. However, when *K* ^+^ channels open this causes membrane depolarization, which closes VDCC, preventing the influx of Ca^2+^, leading to PASMC relaxation, and causing vasodilatation [35].

The voltage-dependent Ca^2+^ channels (VDCC) (especially the dihydropyridine-sensitive L-type channels) have established roles in the regulation of blood pressure; and dihydropyridine Ca^2+^ channel blockers have been clinically used for the treatment of hypertension. The Ca^2+^ influx mechanisms in PASMC via VDCC are influenced by changes in the membrane potential, an electrochemical driving force for Ca^2+^ entry. PASMCs maintain a resting membrane potential (*E*_M_) at about −40 to −60 mV, which is significantly less negative than the equilibrium potential for K^+^ (E_*K*_ is about −85 mV), suggesting that resting *E*_M_ is regulated not only by *K* ^+^ currents, but also background cation (e.g., Na^+^ and Ca^2+^) currents through voltage-independent cation channels and/or nonselective cation channels (i.e., TRP channels). At rest, the membrane is more permeable to *K* ^+^, so background *K* ^+^ currents through voltage-dependent and voltage-independent *K* ^+^ channels are the major determinants for the resting *E*_M_, although Na^+^, Cl^−^, and Ca^2+^ currents also contribute to the regulation of the resting *E*_M_. The activity of various *K* ^+^ channels in the plasma membrane contributes to maintaining the *E*_M_ of PASMC. Inhibition of *K* ^+^ channels causes membrane depolarization, whereas activation of *K* ^+^ channels causes membrane repolarization and hyperpolarization. Among more than 50 different types of *K* ^+^ channels, voltage-gated *K* ^+^ (K_V_) channels are shown to be ubiquitously expressed in smooth muscle cells [37, 38]. When K_V_ channels close, the membrane depolarizes. Following depolarization, voltage-dependent Ca^2+^ channels (VDCCs) open and permeate Ca^2+^ into the cytosol leading to a rise in [Ca^2+^]_cyt_ (Figure 5), thus causing PASMC contraction. 

### 4.2. Store-Operated and Receptor-Operated Ca^2+^ Entry Channels in PASMC

**Figure 6:**
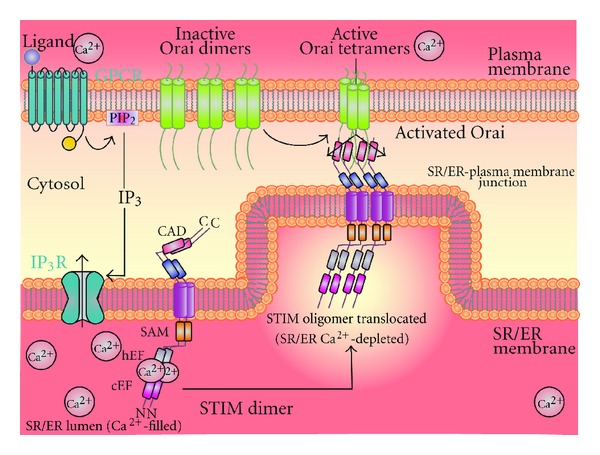
IP_3_ activates voltage-independent Ca^2+^ SOC channels on the plasma membrane, resulting in Ca^2+^ influx into the cell (SOCE). When the SE/ER is filled with Ca^2+^, STIM is evenly dispersed along the membrane and Ca^2+^ is bound to the low-affinity EF hand domain near the N-terminal. Once the IP_3_R is activated by IP_3_, Ca^2+^ levels in the SR/ER decrease. Following detection of Ca^2+^ depletion from the SR/ER store, by the Ca^2+^ binding domain on the N-terminal of STIM found on the SR/ER membrane, Ca^2+^ will dissociate from STIM causing a conformational change that allows for the dimerization and formation of oligomers through interaction of their sterile *α*-motifs (SAMs). Oligomers then translocate to ER/SR-plasma membrane junction where they induce the clustering of SOC channels (e.g., Orai) and stimulate opening of the channel by interaction of the STIM-Orai activating region (SOAR) near the C-terminus on STIM, resulting in an influx of Ca^2+^ [36].

Store-operated Ca^2+^ entry (SOCE), or capacitative Ca^2+^ entry (CCE), is identified as one of the important mechanisms that regulate [Ca^2+^]_cyt_, as addressed by Casteels and Droogmans in early 80s. The groups of channels that mediate SOCE are termed as store-operated Ca^2+^ channels (SOC) [42]. SOC channels are activated by a select group of agonists via cell surface receptors such as G-protein-coupled receptors (GPCR) (Figure 6). Various studies have identified that the stimulation of receptors induces hydrolysis of membrane phosphoinositides by phospholipase C (PLC) yielding the diffusible Ca^2+^-mobilizing messenger inositol 1,4,5-trisphosphate (IP_3_) and diacyl-glycerol (DAG). IP_3_ then binds to IP_3_-receptor to release Ca^2+^ from the internal stores, mainly endoplasmic reticulum (ER) or sarcoplasmic reticulum (SR). The Ca^2+^ release from the internal stores, leading to Ca^2+^ depletion (or significant reduction) from the stores is followed by a stimulated Ca^2+^ entry which is termed as SOCE. The ultimate reason for activation of SOC channels is to maintain long-term cytosolic Ca^2+^ signals and to replenish the depleted ER/SR stores [36, 43]. SOC channels are widely thought to be mediated by Orai (Orai1-3) proteins and different transient receptor potential proteins (TRPs). The characteristics and the components of these channels are discussed below in detail. SOC channels are considered to be most associated with the pathological progression of PAH. Studies have shown that inhibition of SOC expression or activity attenuates PASMC proliferation, indicating the essential role of SOC channels in vascular remodeling and lesions [44].

**Figure 7:**
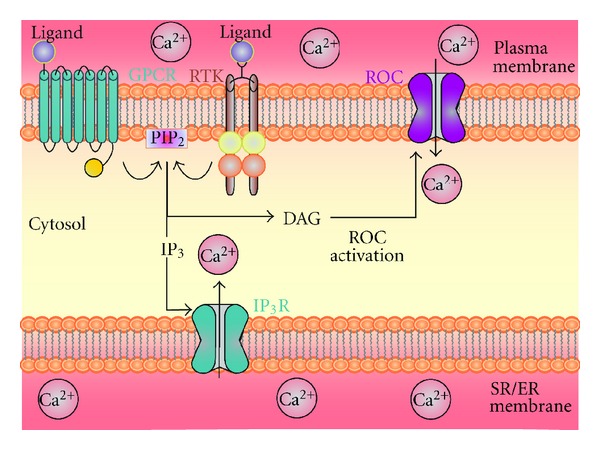
DAG activates voltage-independent Ca^2+^ ROC channels on the plasma membrane, resulting in Ca^2+^ influx into the cell (ROCE). Plasma membrane receptors such as G-protein-coupled receptors (GPCRs) and receptor tyrosine kinases (RTKs) can be activated by various ligands, which then leads to activation of phospholipase C (PLC*γ* or *β*). PLC can then hydrolyze phosphatidylinositol 4,5-bisphosphate (PIP_2_) into inositol 1,4,5-trisphosphate (IP_3_) and diacylglycerol (DAG). DAG remains in the plasma membrane and then activates receptor-operated Ca^2+^ channels (ROCs) also resulting in Ca^2+^ influx.

Receptor-operated channels (ROC) are activated via ligand-mediated activation of receptor tyrosine kinase (RTK) and GPCR. ROC is loosely defined as voltage-independent Ca^2+^ channels that require binding of extracellular ligands to its membrane receptors for activation. Receptor-mediated activation/hydrolysis of PLC releases DAG along with IP_3_ (Figure 7). DAG opens a selective group of plasma membrane- (PM-) localized Ca^2+^ channels leading to Ca^2+^ influx and ultimately a rise in [Ca^2+^]_cyt_, a process referred to as receptor-operated Ca^2+^ entry (ROCE). Although a selective group of channels have been identified to mediate either SOCE or ROCE, dual activation of both SOC and ROC in a given cell type is possible. Ca^2+^ influx mechanisms either via SOCE or ROCE play an important role in the regulation of vascular tone and arterial wall structure [34]. Enhanced SOCE or ROCE in PASMC is evident in patients with PAH and animals with experimental pulmonary hypertension [45]. 

### 4.3. Mediators of SOC

#### 4.3.1. STIM Proteins

**Figure 8:**
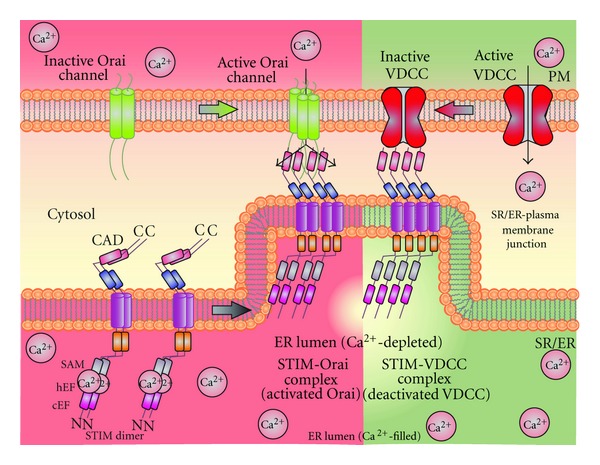
STIM activates SOC and inhibits VDCC: STIM not only interacts with SOC, but recent data show its ability to interact with VDCC in various cell types such as neurons skeletal muscles, and smooth muscle cells. STIM binds to VDCC to inhibit and internalizes VDCC [39–41].

Following the identification of SOC as potential Ca^2+^ influx channels, studies were then geared towards searching for the exact molecular mechanisms by which ER/SR Ca^2+^ store depletion is linked with SOC activation in the plasma membrane. Recently, studies identified stromal interaction molecule (STIM) proteins as the fundamental molecular component of SOC. STIM was originally identified as a cell-surface molecule that mediates cell-stromal interactions [46], before its role in Ca^2+^ signaling was identified. Two isoforms of STIM have been identified, STIM1 and its homologue STIM2. Both STIM1 and STIM2 are expressed in VSMC and endothelial cells. They share similar domain architecture however, the mature STIM2 proteins were shown to be 69 amino acids longer than STIM1 [47]. In recent years, significant progress has been achieved in addressing the role of STIM1 in regulating the PM-Ca^2+^ permeable channels in various cell types, whereas the contribution of STIM2 in mediating SOCE remains controversial and varies in cell types. STIM1 senses the ER/SR Ca^2+^ concentration via its EF hand domain at the N-terminal region [48]. Depletion of ER/SR Ca^2+^, either actively by IP_3_ or passively by inhibiting sarco(endo)plasmic reticulum Ca^2+^-ATPase (SERCA), leads to an unfolding of the EF-hand resulting in a rapid oligomerization with similar domains of neighboring STIM1 molecules [49]. Confirmed findings prove that a mutation within the EF hand showing a constitutively active STIM1 mediates SOCE [50, 51]. Following an unfolding of the EF hand, STIM1 migrates within the ER membrane to regions that are proximal to the plasma membrane and reorganizes into punctae that associate with Ca^2+^ permeable channels to gate or activate these channels [50]. STIM1 interacts with PM components, specifically SOC through their cytoplasmic C termini, which then mediates Ca^2+^ influx. A significant progress has been made in recent years studying the selective gating properties of STIM1 on PM-localized Ca^2+^ permeable channels [52]. Recently, studies in neurons, skeletal muscle cells, and SMC demonstrated that STIM1 also has the ability to interact with VDCC [39–41]. Interestingly, the reason for the association between STIM and VDCC is to inhibit the voltage-dependent Ca^2+^ influx through VDCC and subsequently lead to VDCC internalization (Figure 8) 

STIM2 was initially reported to inhibit SOCE. Furthermore, it was shown that STIM2 coordinates with STIM1 by forming heteromers and serves as a feedback regulator of STIM1-induced SOCE [53]. On the other hand, since STIM2 exhibits low affinity towards Ca^2+^ binding of its EF hand [54], it was proposed to regulate basal cytosol [Ca^2+^]_cyt_ and ER/SR-stored Ca^2+^ concentrations with the possibility of functioning both in store-dependent and store-independent modes, by interaction with calmodulin [53, 55]. Recently, studies from our laboratory showed an important role for STIM2 in mediating SOCE in PASMC. Interestingly an increase in the expression of STIM2 in PASMC was observed in idiopathic pulmonary arterial hypertension (IPAH) patients, which contribute to an augmentation of SOCE and enhancement of PASMC proliferation [56]. More investigation is needed to delineate the potential pathogenic role of STIM2 in the development and progression of pulmonary arterial hypertension.

The two major Ca^2+^ channels that are regulated by STIM and function as SOC are the Orai [36] and TRPC [57, 58] channels. These two types of channels vary based on their biophysical properties. Orai channels mediate the highly Ca^2+^-selective, inward rectifying Ca^2+^ release-activated Ca^2+^ current termed as *I*_CRAC_ [59], whereas transient TRP channels mediate a nonselective, Ca^2+^ permeable current termed as *I*_SOC_ [60]. A significant progress has been made in recent years in addressing the molecular composition of SOC and the fundamental mechanisms by which these channels are activated in vascular smooth muscle cells. In this paper, we focus to update on the current knowledge of Ca^2+^ channels associated with vascular smooth muscles.

#### 4.3.2. Orai-STIM Signaling in Pulmonary Vasculature

*I*_CRAC_ were the first and best-characterized SOC current. Until recently, CRAC currents were considered to be associated mostly with cells of the hematopoietic lineage. Studies in recent years have identified the existence of these currents in nonexcitable cells such as vascular smooth muscle and endothelial cells [61]. The pore forming channels that were unequivocally proven to mediate *I*_CRAC_ are Orai proteins [36, 62, 63]. Orai are tetraspanning membrane proteins that play a significant role in mammalian cell morphology and motility. Three isoforms of Orai family proteins (Orai1-3) have been identified, which display notable differences in their features despite a high degree of sequence similarity. Among the three isoforms, Orai1 is the most potent ion pore forming subunit in most cells, and its depletion has the highest impact on SOCE in SMC [64–66]. The expression levels of Orai1 in SMC of arterial sections were seen to be relatively less [66, 67] compared to cells from coronary [68] or carotid arteries [67]. On the contrary, an increase in the detection levels of Orai1 was observed in SMC from injured arteries, either by physical or metabolic means [66–68]. Furthermore, findings from our laboratory also demonstrated an increase in the expression of STIM1 and Orai1 in SMC induced by platelet-derived growth factor (PDGF), through mammalian target of rapamycin (mTOR) pathway [69]. Additionally, the inhibition of the Akt/mTOR pathway significantly suppressed the proliferation rate of PASMC. Our unpublished data also show that an increase in the expression of Orai proteins in proliferating SMC occurs compared to native SMC maintained in a contractile phenotype. In addition, levels of protein and mRNA of Orai are expressed in the A10 cell line (model system for proliferating vascular smooth muscle cells) [70], and in *in vivo* injured arteries (i.e., monocrotaline- (MCT-) induced PAH) [67]. These observations clearly substantiate the above findings that the expression of Orai proteins may play an important role in the transition between the proliferative phenotype and the contractile phenotype either *in vitro* or *in vivo*.

STIM proteins play a central role in activating CRAC current mediated by Orai. Evidence showing the generation of CRAC currents when Orai1 was expressed with STIM1 [71], but not Orai1 alone, clearly ascertained Orai-STIM signaling. Furthermore, when the charged residues in the transmembrane domains were mutated, the ionic selectivity of Orai channels was modified [62, 72]. Studies intended to address how STIM1 opens the Orai channels and identified the binding of STIM1, STIM2, and/or other potential proteins with the intracellular N- and C-termini of Orai1. Upon store Ca^2+^ depletion, STIM1 coclusters with Orai and forms punctae, representing a fine stoichiometry between Orai, STIM, and other possible molecular candidates in determining the magnitude of Ca^2+^ entry. It was identified that a minimal domain of STIM1 is sufficient to activate CRAC channels (named SOAR for STIM1-Orai activating region). Cross-linking studies indicate that Orai1 proteins form dimers in resting state [36]. However, once activated four molecules of Orai1 form an active pore and mediate a sustained influx of Ca^2+^ into the cytosol. In addition to Orai1, Orai2 was also identified to yield Ca^2+^-selective *I*_CRAC_ currents, while Orai3 resulted in a small and slowly developing *I*_CRAC_ [71, 73]. Although the Orai-mediated CRAC currents are quite well understood in lymphocytes and certain nonexcitable cells, a definitive role of Orai in VSMC and endothelial cells is still not clear. In lung microvessel endothelial cells, knockdown or overexpression of Orai1 proteins showed no significant effect on SOCE [74]. Since Orai proteins are present in endothelial cells, more studies are warranted to define the role of Orai in SOC currents.

#### 4.3.3. TRPC-STIM Signaling in Pulmonary Vasculature

TRP channels have also been shown to mediate SOC. It is generally considered to be important signal transducers for agonist-mediated vascular contractility. The role of TRP channels in Ca^2+^ influx mechanisms was first discovered in *Drosophila*. Most TRP channels are nonselective for monovalent and divalent cations with Ca^2+^: Na^+^ permeability ratio <10 [75]. The first mammalian TRP protein identified in humans and mice was TRPC1. Since its identification, a number of TRP proteins have been found. Among these TRP families of proteins are TRPC, TRPV, TRPA, TRPM, which are closely related to each other, while TRPP and TRPML are distantly related subfamilies. The canonical TRP and (TRPC) subfamily is comprised of seven members (TRPC1-7). Initially, TRPC channels were thought to be mainly involved in mediating ROCE mechanisms. However, in recent years, substantial evidence supports the significant role for TRP proteins in the conduction of Ca^2+^ entry during SOCE. Using approaches such as overexpression and knockdown, several members of the TRPC family are reported to be activated by Ca^2+^ store depletion. However, it is evident from various investigations that TRPC proteins serve as an SOC based on its expression levels and its tendency to associate with other proteins. Interestingly, it was shown that TRPCs which serve as an SOC at lower expression may not be sensitive to store depletion but may be activated by PLC or its metabolites [76]. There is also evidence that TRPC subfamily of channels may function both as SOCE or ROCE in the same cell type depending on their levels of expression [77]. 

Several studies have confirmed the existence of TRPC channels in various vascular preparations [74, 78, 79]. Among the members of TRPC family of proteins, TRPC1, TRPC3, TRPC4 and TRPC6 have been studied extensively showing their abundant expression levels in pulmonary artery smooth muscle and intralobar PASMC [45, 80–82]. Most importantly, it was shown that the expression of TRPC1 and TRPC6 is upregulated in PASMC of hypoxia-induced pulmonary hypertension, a cause for an increase in SOCE and ROCE [83, 84]. Furthermore, the resulting elevated resting intracellular Ca^2+^ levels in PASMC were shown to augment the resting tension of pulmonary arteries of chronic hypoxic rats [83]. Recently, a study by Liu et al. also demonstrated the association of TRPC1-dependent SOCE in MCT-induced contraction in pulmonary arteries, a representative model of PAH in mice [85]. The TRPC1 channel can be activated through ET-1-induced PKC stimulation [3, 86, 87]. Activation of SOC increases [Ca^2+^]_cyt_ and thus produces vasoconstriction, promotes cell cycle progression, and ultimately leads to PASMC proliferation and vascular remodeling [34, 60, 86, 88, 89]. 

TRPCs (TRPC1, TRPC3, TRPC4, TRPC5, and TRPC6, but not TRPC7) were shown to be regulated by STIM1 in various cell types. STIM1 binds to and appears to directly regulate TRPC1, TRPC4, and TRPC5, as they are sensitive to store depletion. On the other hand, TRPC3 and TRPC6 may be activated indirectly and involve STIM1-dependent heteromultimerization of TRPC1-TRPC3 and TRPC4-TRPC6, in which TRPC1 and TRPC4 present STIM1 to TRPC3 and TRPC6, respectively [90]. STIM1-TRPC1/4 interaction is shown to mediate the STIM1 ERM domain. Association of STIM1 and TRPC1 following store depletion was shown in VSMC [91]. Interestingly, only partial interaction between STIM1 and TRPC1 was observed depicting that these two proteins have independent functions in VSMC [91]. It may also be because TRPC1 forms homotetramer or heterotetramer with other TRPC channels, or other transmembrane proteins to form SOC. Recently, studies using lung microvessel endothelial cells from TRPC4^−/−^ mice showed an intermittent role of STIM1-TRPC signaling in mediating SOCE and thereby affecting vascular functions [74].

#### 4.3.4. STIM1-TRPC-Orai Signaling

TRPC and Orai1 clearly differ from each other with their channel properties and functions when activated by STIM1. Although both are gated by STIM1, it was believed that STIM1 gates TRPC and Orai channels by separate mechanisms [92]. Since Orai, TRPC channels, and STIM1 are found to be expressed and function in the same cells, it was believed that these proteins depend on the activity of each other. Several studies suggested that Orai1 and TRPC channels may interact and contribute to Ca^2+^ influx via SOC [93]. Using transgene expression of low levels of Orai1 in cells expressing TRPC1/3/6 studies showed an enhanced SOC activity [94, 95]. Furthermore, Orai1 was shown to interact with C and N termini of TRPC channels [94]. In a separate group of studies, knockdown of endogenous Orai1 or expressing functionally defective mutant (Orai1-R91W, E10Q) blocked SOCE induced by TRPC1-STIM1 overexpression [93, 96]. Formation of a functional TRPC1-STIM1-Orai1 complex upon store depletion highlights the critical contribution of this dynamic assembly to mediate SOCE. Recently, Cioffi et al. [97] demonstrated that Orai1 associates with TRPC4, and forms a functional channel complex. In addition, they also showed that Orai1-TRPC4 interaction may be involved in controlling TRPC1/4 activation and channel permeation characteristics. On the other hand, few studies showed that TRPC-knockout mice lacked functional compensation by the residual Orai and STIM1 proteins [74]. In fact, overexpression of STIM1 or Orai1 proteins in TRPC4^−/−^ lung endothelial cells did not induce STIM1 puncta formation and SOCE [74]. Thus, this prevailing evidence suggests that native SOCE components and signaling may vary in different cell types based on the specific physiological functions that regulate SOCE.

### 4.4. Mediators of ROC

Unlike SOCE, ROCE is activated independent of store depletion or Ca^2+^ release from the intracellular stores. Several members of TRPC family proteins are identified to be involved in ROCE mechanisms in PASMC and play a role in pathogenesis of familial PAH and IPAH. In PASMC derived from IPAH patients, the expression levels of TRPC3 and TRPC6 were shown to be upregulated compared in normal subjects and patients with secondary pulmonary hypertension [98, 99]. Studies from our laboratory have also shown an increase in TRPC3 isoforms in addition to TRPC6 in PASMC of IPAH patients [100]. Interestingly, it was found that deletion of TRPC6 caused an upregulation of TRPC3 expression in some tissues [101], which may be a compensatory mechanism to mediate ROCE. TRPC6 channels were shown to critically contribute to early phase hypoxia-induced pulmonary vasoconstriction and IPAH [83, 102]. The role of TRPC6 was primarily assigned as a channel that regulates Ca^2+^ entry and induces contraction in vascular smooth muscle. TRPC6 is a member of the DAG-sensitive TRPC subfamily, which has been shown to increase [Ca^2+^]_cyt_ in a membrane-delimited fashion. TRPC6 is insensitive to PLC-IP_3_ activation at the cellular level [103]. As its nature to be sensitive for DAG, it was hypothesized that an increase in the DAG may induce hypoxia-induced pulmonary vasoconstriction. This hypothesis was mainly derived from the earlier findings that hypoxia induces an accumulation of DAG at the PASMC membrane [104]. Furthermore, a single-nucleotide polymorphism (SNP) −254(C-G) was identified in TRPC6 regulatory regions of 268 IPAH patients. Exploring the functional effects of this SNP strikingly showed that it generates a nuclear factor *κ*B (NF-*κ*B) response element in the TRPC6 regulatory regions [102]. Additionally, using promoter mutations that lead to TRPC6 overexpression and using targeted gene disruption approaches (TRPC6^−/−^) [105–107], the indispensable role of TRPC6 in regulating the tone and diameter of pulmonary arteries was demonstrated [108]. Furthermore these findings link NF-*κ*B to the nuclear translocation of NF-*κ*B upregulated expression of TRPC6, and thereby enhancing Ca^2+^ influx mechanism in PASMC from IPAH patients. In addition, a direct link between NF-*κ*B and inflammation also supports the hypothesis that some of the pathophysiology of PH involves an inflammatory response [109]. More research is in need to identify the factors essential for NF-*κ*B activation associated with PH. 

### 4.5. TRPV and TRPM in Pulmonary Vasculature

Besides TRPC channels, very little is known about other TRP channel subfamilies in pulmonary vasculature. Prevailing investigations on other TRP channels identified TRPV (Vanilloid) and TRPM (Melastatin) channels in PASMC, rat intralobar pulmonary arteries, and also in aorta. The mRNA of TRPV1, TRPV2, TRPV3, TRPV4, and TRPV6 of the TRPV family and TRPM2, TRPM3, TRPM4, TRPM7, and TRPM8 of the TRPM family were detected in pulmonary arteries. 

TRPV channels are widely expressed in various cell types and are nonselective cation channels that possess high Ca^2+^ permeability. These channels can be activated by capsaicin and a diverse range of biological stimuli, including acid, temperature changes, osmolarity, mechanical stress, intracellular Ca^2+^ concentrations, and various inflammatory mediators [110]. TRPV family of channels are shown to be upregulated by chronic hypoxia and may execute effects related to cell proliferation by forming a heterotetrameric SOC channels [111]. Among the TRPV channel subfamily, TRPV4 predominantly is expressed in PASMCs and other smooth muscle cells and is involved in mediating SOCE [111–113]. Activation of TRPV4 in SMC causes membrane hyperpolarization and dilation of cerebral arteries through large-conductance Ca^2+^-activated *K* ^+^(BK_Ca_) channels [114]. This study also suggests that Ca^2+^ influx via TRPV4 may stimulate BK_Ca_ currents and may hyperpolarize arterial smooth muscle cells, promoting arterial relaxation [115]. In addition, TRPV1 and TRPV2 were shown to have relatively low expression and may be involved in evoking Ca^2+^ response in PASMC [114]. These responses were completely abolished when the Ca^2+^ was removed or when Ni^2+^ was added to the bath solution [116] but unaffected by nifedipine. Studies suggest that capsaicin, a TRPV1 agonist, may act directly on smooth muscle cells to elicit vasoconstriction [117]. Similar to TRPV4, TRPV1-dependent Ca^2+^ entry was also demonstrated to mediate hypoxia-induced proliferation of PASMC [111]. TRPV2 expression was detected in mouse aortic, mesenteric artery and basilar artery smooth muscle cells [118]. TRPV2 channels were identified to induce nonselective cation currents and Ca^2+^ influx in mouse aortic smooth muscle cells [118] and recently in PASMC [119].

TRPM family of proteins is comprised of eight members (TRPM1-8). Several studies report that TRPM8 is the most highly expressed TRPM mRNA in pulmonary artery smooth muscles, while TRPM4 and TPRM7 are present at relatively low levels [113, 120]. Activation of these channels with specific agonists induced Ca^2+^ elevations, indicating their functional relevance in vascular smooth muscles. TRPM4 channels are a crucial mediator of pressure-induced vascular smooth muscle membrane depolarization and vasoconstriction. TRPM4 is identified as a Ca^2+^-activated monovalent cationic channel [121, 122]. TRPM4 is impermeable to Ca^2+^, but studies using inside-out patch clamp technique demonstrated that TRPM4 channels can be induced by the higher levels of intracellular Ca^2+^ (1–10 *μ*M) in native smooth muscle cells [123]. Furthermore, these findings also revealed that the resulting raise in [Ca^2+^]_cyt_ may presumably induce BK_Ca_ (like TRPV4) and regulate membrane potential depolarization. Thus, TRPM4-mediated depolarization may support Ca^2+^ influx. These findings were further substantiated by diminishing the expression of TRPM4 in the cerebral vasculature, which resulted in a loss of autoregulation of cerebral blood flow in response to changes in perfusion pressure [124]. TRPM7 was shown to mediate Mg^2+^ influx in response to angiotensin II (Ang II), which in turn may be required for vascular SMC proliferation [125].

TRPM8 is identified as a cold-sensitive Ca^2+^ permeable channel expressed in the vasculature. Similar to TRPV4 (as discussed above), TRPM8 is capable of evoking Ca^2+^ influx in PASMC and also in aortic SMC. On the other hand, studies have reported that TRPM8 may participate in Ca^2+^ signaling through a phosphatidylinositol 4,5-bisphosphate (PIP_2_-) dependent mechanisms independent of cold stimulus. This suggests that TRPM8 may also be activated by receptor-dependent mechanisms, which modulates PIP_2_ synthesis and hydrolysis [126]. Few other studies suggest that TRPM8 agonists, menthol and icillin, release Ca^2+^ from ryanodine receptors and hyperpolarize the smooth muscle PM resulting in arterial dilation [127]. On the contrary, findings by Mahieu et al. reported that menthol-induced intracellular Ca^2+^ release is independent of TRPM8 [128]. These confounding reports result in unclear issues. Adding to the mystery, TRPM8 currents have not been reported in SMC, and the endogenous activators of this channel are not known. 

## 5. Conclusion and Future Directions 

In order to understand the pathogenic mechanisms of PAH, it is important to consider Ca^2+^ regulation in pulmonary vasoconstriction and PASMC proliferation. Two mechanisms, voltage-dependent and voltage-independent Ca^2+^ influx pathways, regulate [Ca^2+^]_cyt_. Variations in the expression and function of the ion channels necessary for Ca^2+^ influx are key features in the development and pathogenesis of PAH. In PAH, a rise in [Ca^2+^]_cyt_ in PASMC due to enhanced SOCE/ROCE (due to upregulated SOC/SOC components) and voltage-dependent Ca^2+^ entry in PASMC from IPAH patients also activates many signal transduction proteins (e.g., CaMK, PKC, and MAPK) and transcription factors (e.g., AP-1, NFAT, CREB, and NF-*κ*B) thus influencing gene expression and promoting cell proliferation. Experiments have therefore shown that Ca^2+^ channels and significant signaling pathways are possible sites for therapeutic targets. 

Future experiments detailing the specific downstream effects of enhanced SOCE and ROCE will shed light on important therapeutic strategies for PAH. More specifically, understanding the role of other proteins such as transcription factors, signal transduction proteins, and nuclear transporters and their effect on vasoconstriction and proliferation could provide future pharmacological blockades of SOC/ROC. Additionally, downregulation of these proteins could reveal therapeutic effects for PH. Application of new experimental methods along with further use of well-established methods is crucial to the advancement of the field of pulmonary hypertension. 

## Figures and Tables

**Table 1:** WHO classification of pulmonary hypertension (Dana Point, 2008) (reprinted from [5]).

(I) Pulmonary arterial hypertension (PAH)
(1) Idiopathic PAH
(2) Heritable
(i) BMBR2
(ii) ALK1, endoglin (with or without hereditary hemorrhagic telangiectasia)
(iii) Unknown
(3) Drug and toxin induced
(4) Associated with
(i) Connective tissue diseases
(ii) HIV infection
(iii) Portal hypertension
(iv) Congenital heart diseases
(v) Schistosomiasis
(vi) Chronic hemolytic anemia
(5) Persistent pulmonary hypertension of the newborn
(II) Pulmonary hypertension due to left heart disease
(1) Systolic dysfunction
(2) Diastolic dysfunction
(3) Valvular disease
(III) Pulmonary hypertension due to lung diseases and/or hypoxia
(1) Chronic obstructive pulmonary disease
(2) Interstitial lung disease
(3) Other pulmonary diseases with mixed restrictive and obstructive patterns
(4) Sleep-disordered breathing
(5) Alveolar hypoventilation to high altitude
(6) Developmental abnormalities
(IV) Chronic thromboembolic pulmonary hypertension (CTEPH)
(V) PH with unclear multifactorial mechanisms
(1) Hematologic disorders: myeloproliferative disorders and splenectomy
(2) Systemic disorders: sarcoidosis, pulmonary Langerhans cell histiocytosis, lymphangioleiomyomatosis, neurofibromatosis, and vasculitis
(3) Metabolic disorders: glycogen storage disease, Gaucher disease, and thyroid disorders
(4) Others: tumoral obstruction, fibrosing mediastinitis, and chronic renal failure (on dialysis)

**Table 2:** Functional classification of pulmonary hypertension (reprinted from [6] with permission from the American College of Chest Physicians).

(A) New York Heart Association functional classification
(i) Class 1: no symptoms with ordinary physical activity
(ii) Class 2: symptoms with ordinary activity; slight limitation of activity
(iii) Class 3: symptoms with less than ordinary activity; marked limitation of activity
(iv) Class 4: symptoms with any activity or even at rest
(B) WHO functional assessment classification
(i) Class I: patients with PH but without resulting limitation of physical activity; ordinary physical activity does not cause undue
dyspnea or fatigue, chest pain, or near syncopy
(ii) Class II: patients with PH resulting in slight limitation of physical activity; they are comfortable at rest; ordinary physical
activity causes undue dyspenea or fatigue, chest pain, or near syncope
(iii) Class III: patients with PH resulting in marked limitation of physical activity; they are comfortable at rest; less than ordinary
activity causes undue dyspenea or fatigue, chest pain, or near syncope
(iv) Class IV: patients with PH with inability to carry out any physical activity without symptoms; these patients manifest signs of right
-heart failure; dyspnea and/or fatigue may even be present at rest; discomfort is increased by any physical activity
